# COVID-19 testing acceptability and uptake amongst the Rohingya and host community in Camp 21, Teknaf, Bangladesh

**DOI:** 10.1186/s13031-020-00322-9

**Published:** 2020-11-11

**Authors:** Catherine R. McGowan, Nora Hellman, Sayem Chowdhury, Abdul Mannan, Katherine Newell, Rachael Cummings

**Affiliations:** 1 Humanitarian Public Health Technical Unit, Save the Children UK, 1 St John’s Lane, London, EC1M 4AR UK; 2 Department of Public Health, Environments & Society, London School of Hygiene & Tropical Medicine, 15-17 Tavistock Place, London, WC1H 9SH UK; 3 Save the Children Bangladesh, Rohingya Response, Cox’s Bazar, Bangladesh

**Keywords:** COVID-19, Infectious diseases, Forcibly Displaced Myanmar Nationals, FDMN, Sentinel testing, Rohingya, Acceptability, Testing uptake

## Abstract

Facility-based sentinel testing for COVID-19 was implemented in May 2020 to monitor the prevalence of COVID-19 amongst the Rohingya and host community in Cox’s Bazar, Bangladesh. In response both to low uptake of testing across all camps, and rumours of an outbreak of an influenza-like illness in May/June 2020, the International Organization for Migration (in partnership with ACAPS) undertook a qualitative study to collect accounts from the Rohingya relating to testing and treatment, and to explore the possibility that what was thought to be an outbreak of influenza may have been COVID-19. The report provided rich descriptions of the apprehension around testing and offered some clear recommendations for addressing these. We developed a testing ‘script’ in response to these recommendations, deploying it alongside a survey to determine reasons for declining a test. We compared testing uptake before deploying the testing script, and after (controlling for the total number of consultations), to generate a crude measure of the impact of the script on testing uptake. We coded reasons for declining a test thematically, disaggregated by status (Rohingya and host community) and sex. Despite the small sample size our results suggest an increase in testing uptake following the implementation of the script. Reasons provided by patients for declining a test included: 1) fear, 2) the belief that COVID-19 does not exist, that Allah will prevent them from contracting it, or that their symptoms are not caused by COVID-19, 3) no permission from husband/family, and 4) a preference to return at a later time for a test. Our findings largely mirror the qualitative accounts in the International Organization for Migration/ACAPS report and suggest that further testing amongst both populations will be complicated by fear, and a lack of clarity around testing. Our data lend force to the recommendations in the International Organization for Migration/ACAPS report and emphasise that contextual factors play a key role and must be considered in designing and implementing a health response to a novel disease.

## Introduction

There are currently 860,494 Rohingya in Cox’s Bazar, Bangladesh [1]. In addition, there are an estimated 335, 900 individuals in the host community [2]. Save the Children International (SCI) currently operates one Primary Health Care Centre (PHCC), eight health posts, and a newly constructed Severe Acute Respiratory Infection Isolation and Treatment Centre (SARI ITC) to serve both Rohingya and host communities. In response to growing concern about the potential for COVID-19 to cause significant morbidity and mortality, WHO and the Bangladesh Ministry of Health established eight sentinel testing sites for COVID-19, including the SCI PHCC. The initial protocol (dated 24 May 2020) required that anyone presenting at a sentinel site with any symptom of acute respiratory illness (ARI) be offered a COVID-19 test. Patients consenting to a test would be asked to isolate at the sentinel site until the test result was available (approximately 48 h). On 11 June 2020, in response to low testing uptake, the Bangladesh Refugee Relief and Repatriation Commissioner (RRRC) decided to allow patients to return home while their test was being processed; however, uptake of testing for COVID-19 at the PHCC (and across all sentinel sites) remained low [3].

In response to low uptake of testing across all camps - as well as reports of an outbreak of an influenza-like illness in the camps in May/June and the absence of a corresponding increase in primary healthcare consultations - the International Organization for Migration (IOM), in partnership with ACAPS, undertook a qualitative study to collect accounts from Rohingya around testing and treatment, and to explore the possibility that what was thought to be an outbreak of influenza may have been COVID-19. The report, which was based on interviews collected by Rohingya researchers between 25 May and 25 June 2020, explored reasons for the low uptake of testing. The report suggested that, “[a] general consensus seemed to have formed in the community not to test and to avoid seeking treatment” ([4], p. 2). The report further highlighted that the Rohingya’s reasons for not engaging in testing were many and included: rational concerns about being asked to remain at a health facility for two days to await test results, lack of clarity about the testing process (including why they were being asked to undergo testing), fear of lockdown of entire sub-blocks as a result of a positive test, concerns about incidents involving disclosure of patient details and test results to the public, and a lack of clarity on the benefits of testing [4].

**Fig. 1:**
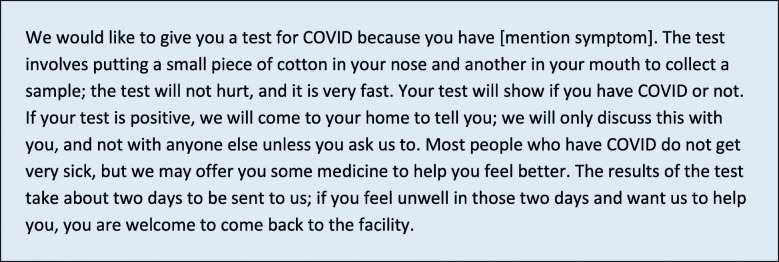
Standard COVID-19 testing script

Therefore, to increase testing uptake, and understand testing acceptability, we created a script (Fig. 1) – addressing concerns raised in the IOM/ACAPS report - for health care workers (HCW) to read to patients. In addition, we asked HCWs to record some basic demographic information about patients who were offered a test and, if they declined, to document the reason for refusal. As the report cautioned against pressuring patients for information, we designed the survey to be brief and unintrusive.

## Methods

The testing script was developed by the SCI Public Health Director and the Monitoring, Evaluation, Accountability and Learning (MEAL) Manager for the Cox’s Bazar COVID-19 response. With the support of the PHCC Medical Officer and the Laboratory Technician we trained PHCC staff on communicating the content of the testing script (both in Bangla and Chittagonian) and on the collection of the survey data. We deployed our testing script and survey using Kobo Toolbox (Harvard Humanitarian Initiative, Cambridge, MA). The survey was designed to collect age, status (Rohingya or host community), and whether or not the patient had agreed to a test. We also collected information on the acceptability of testing by asking those who had declined a test to indicate their reason for refusal. HCWs administered the test with support from a translator if required. No fields were mandatory, and staff were requested not to put pressure on patients to explain their decision. We defined ‘uptake’ as the proportion of tests administered compared to the number of tests offered. We defined ‘acceptability’ as the qualitative justification for declining a test. We coded the reasons for declining a test thematically. We used routinely collected data for total consultations, combined with testing data, to determine change in uptake.

## Results

We deployed the survey (see Table 1) between 9 July and 21 October 2020. Of the 222 patients who were offered a COVID-19 test 60% (*n* = 133) were women, 61% were Rohingya (*n* = 136); just under half accepted the offer of a test (*n* = 113, 51%). Of the 109 patients who refused a test, 26 (24%) declined to provide a reason for doing so. The four most commonly cited reasons (comprising 88% of the responses) were: 1) fear (*n* = 27; *n* = 21 female; *n* = 23 Rohingya), 2) does not believe in COVID-19, believes Allah will protect them from contracting COVID-19, or does not believe symptoms are due to COVID-19 (*n* = 25; *n* = 17 female, *n* = 18 Rohingya), 3) no permission from husband/family (*n* = 11; n = 11 female, n = 11 Rohingya), and patient will come back later (*n* = 10; *n* = 4 female, n = 4 Rohingya).

**Table 1:** Demographic characteristics and results

Age		
Mean (SD)	31 (14.58)	
Median	27	
Range	14–100	
Sex		
Female	133	60%
Male	89	40%
Status		
Rohingya	136	61%
Host community	86	39%
Agreed to test		
Yes	113	51%
No	109	49%
Reason (*n* = 83)		
Fear	27	33%
Does not believe in COVID-19; believes Allah will protect her/him from COVID-19; does not believe s/he has symptoms of COVID-19	25	30%
Husband/family will not allow; does not have permission	11	13%
Will come back later	10	12%
Other (e.g. has already had test…)	10	12%

**Fig. 2:**
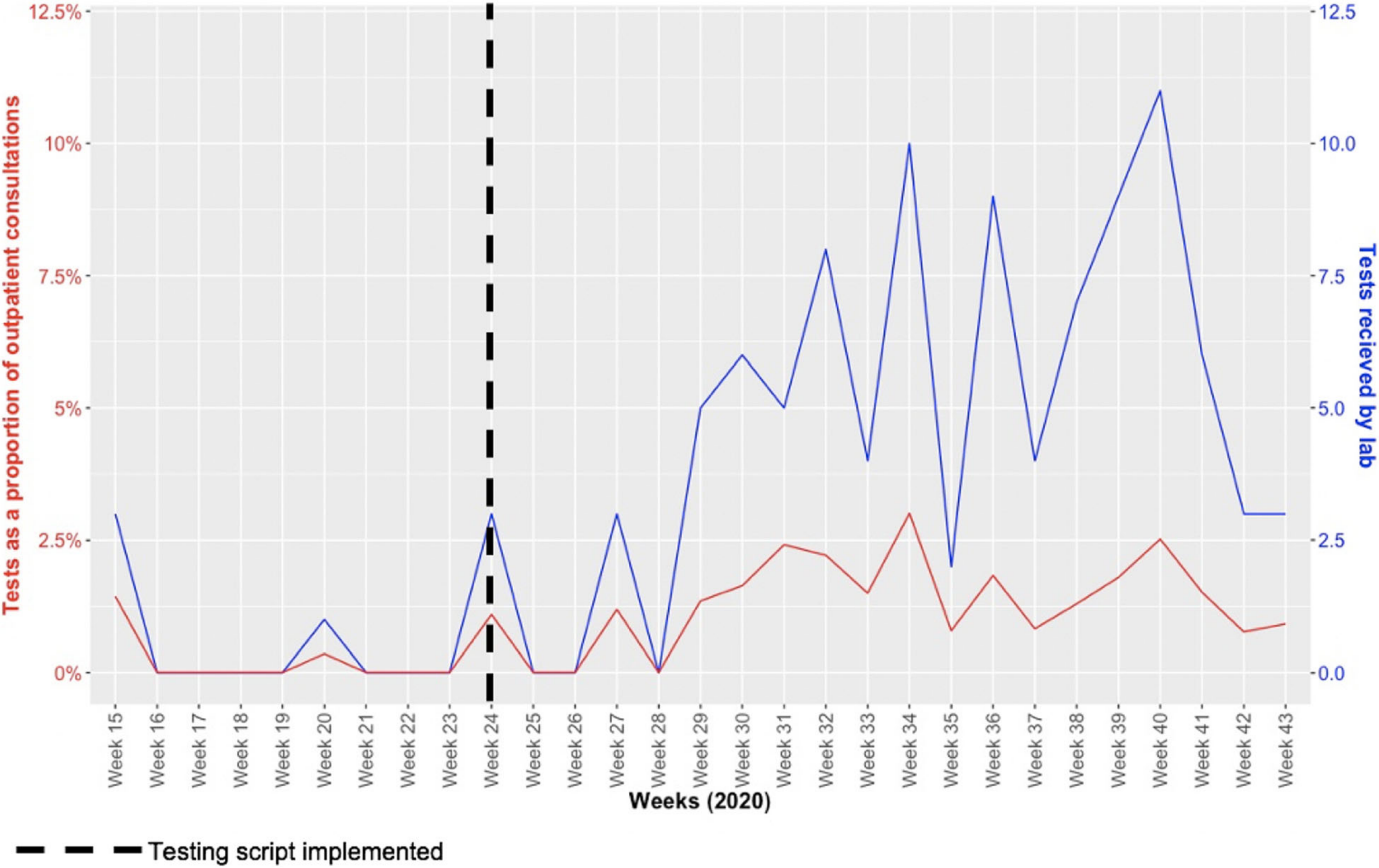
Tests as a proportion of weekly outpatient consultations and tests received by the lab

Univariate analysis was performed using the chi-square test of independence to assess the hypothesis of association between the observed frequencies in the categorical variables of script use and test consent status in new consultations (see Fig. 2). We considered a *p*-value of < 0.05 as statistically significant. The association between script use and test consent status was statistically significant, *X*^2^ (1, *N* = 6180) = 49.2 *p* = < 0.001 with patients offered a testing script more likely to consent to COVID-19 testing.

## Discussion

Our survey demonstrates that though our efforts to provide clarity and the necessary assurances around testing have significantly improved uptake, only half of eligible patients were willing to accept the offer of a COVID-19 test. Many patients declined to provide a justification for refusing a test; however, the justifications that were provided suggest: fear, poor understanding of COVID-19 susceptibility and symptoms, lack of an urgency around testing, lack of understanding of the benefits of testing (or low appeal of the benefits when compared to the risks), and an inability to consent to a test without permission from a family member (most often a husband). It is worth noting that responses indicating that patients did not believe they had COVID-19, or did not believe that their symptoms were indicative of COVID-19, may reflect the assumption that the outbreak had happened earlier in the year as suggested by the IOM/ACAPS report [4]. We also demonstrate that addressing Rohingya concerns by explaining the purpose of test improved uptake.

A key limitation to our study is its small sample size which may have increased the probability of Type 2 error. Furthermore, we accept that a range of responses (e.g. having already had a test, needing permission from husband/family, or promises to return later for a test) may proxy for fear or disinterest. However, the responses to our survey, regardless of their relative proportion and lack of statistical power, are notable in that they lend force to the findings of the qualitative accounts detailed in the IOM/ACAPS report which found that the Rohingya population went to great lengths to avoid being tested owing to historical fears of mistreatment by authorities and health actors, to more recent accounts of “…negative, stigmatising, undignified, and difficult experiences around the testing and treatment process” [4, 5]. Finally, we have demonstrated that uptake of testing increased once we began providing standard, scripted assurances around the confidentiality of test results, and information addressing some of the lack of clarity around the testing protocol.

## Conclusion

The IOM/ACAPS report cautions that, “[a] general consensus seems to have formed in the community not to test” and that “applying more pressure or trying to ‘investigate’ events may frighten people, resulting in their hiding or fleeing from such efforts” ([4], p. 2). Furthermore, various breaches of confidentiality early in the response have compounded mistrust around testing [4]. Further testing within this population to evidence the trajectory of the outbreak, and to establish if accounts of a rise in ARIs in the camps in May–June may have been COVID-19, would likely require a large-scale sero-survey. However, our survey suggests that challenges to testing are likely to persist unless considerable efforts are made to address rational fears around testing relating largely to the complex history of the Rohingya population, and to more proximal and immediate fears of lock-down or disclosure of test results. High vaccine uptake in the camps suggests that Rohingya/host populations may not be averse to sero-surveys provided their benefits are clearly communicated. Our experience of low uptake of COVID-19 testing amongst PHCC patients in Camp 21 underlines the importance of considering contextual factors when designing and implementing a health response, especially to a novel disease.

## Data Availability

Not applicable.
